# Multi-omics to predict acute radiation esophagitis in patients with lung cancer treated with intensity-modulated radiation therapy

**DOI:** 10.1186/s40001-023-01041-6

**Published:** 2023-03-19

**Authors:** Xiaoli Zheng, Wei Guo, Yunhan Wang, Jiang Zhang, Yuanpeng Zhang, Chen Cheng, Xinzhi Teng, Saikit Lam, Ta Zhou, Zongrui Ma, Ruining Liu, Hui Wu, Hong Ge, Jing Cai, Bing Li

**Affiliations:** 1 Department of Radiation Oncology, The Affiliated Cancer Hospital of Zhengzhou University & Henan Cancer Hospital, Zhengzhou, China; 2 Department of Health Technology and Informatics, The Hong Kong Polytechnic University, Hong Kong SAR, China; 3 Department of Interventional Therapy, Henan Provincial People’s Hospital, Zhengzhou, China

**Keywords:** Acute radiation esophagitis, Lung cancer, Radiomics, Dosiomics, Machine learning, Radiotherapy

## Abstract

**Purpose:**

The study aimed to predict acute radiation esophagitis (ARE) with grade  ≥ 2 for patients with locally advanced lung cancer (LALC) treated with intensity-modulated radiation therapy (IMRT) using multi-omics features, including radiomics and dosiomics.

**Methods:**

161 patients with stage IIIA−IIIB LALC who received chemoradiotherapy (CRT) or radiotherapy by IMRT with a prescribed dose from 45 to 70 Gy from 2015 to 2019 were enrolled retrospectively. All the toxicity gradings were given following the Common Terminology Criteria for Adverse Events V4.0. Multi-omics features, including radiomics, dosiomics (including dose−volume histogram dosimetric parameters), were extracted based on the planning CT image and three-dimensional dose distribution. All data were randomly divided into training cohorts (N = 107) and testing cohorts (N = 54). In the training cohorts, features with reliably high outcome relevance and low redundancy were selected under random patient subsampling. Four classification models (using clinical factors (CF) only, using radiomics features (RFs) only, dosiomics features (DFs) only, and the hybrid features (HFs) containing clinical factors, radiomics and dosiomics) were constructed employing the Ridge classifier using two-thirds of randomly selected patients as the training cohort. The remaining patient was treated as the testing cohort. A series of models were built with 30 times training–testing splits. Their performances were assessed using the area under the ROC curve (AUC) and accuracy.

**Results:**

Among all patients, 51 developed ARE grade  ≥ 2, with an incidence of 31.7%. Next, 8990 radiomics and 213 dosiomics features were extracted, and 3, 6, 12, and 13 features remained after feature selection in the CF, DF, RF and DF models, respectively. The RF and HF models achieved similar classification performance, with the training and testing AUCs of 0.796 ± 0.023 (95% confidence interval (CI [0.79, 0.80])/0.744 ± 0.044 (95% CI [0.73, 0.76]) and 0.801 ± 0.022 (95% CI [0.79, 0.81]) (*p* = 0.74), respectively. The model performances using CF and DF features were poorer, with training and testing AUCs of 0.573 ± 0.026 (95% CI [0.56, 0.58])/ 0.509 ± 0.072 (95% CI [0.48, 0.53]) and 0.679 ± 0.027 (95% CI [0.67, 0.69])/0.604 ± 0.041 (95% CI [0.53, 0.63]) compared with the above two models (*p* < 0.001), respectively.

**Conclusions:**

In LALC patients treated with CRT IMRT, the ARE grade  ≥ 2 can be predicted using the pretreatment radiotherapy image features. To predict ARE, the multi-omics features had similar predictability with radiomics features; however, the dosiomics features and clinical factors had a limited classification performance.

**Supplementary Information:**

The online version contains supplementary material available at 10.1186/s40001-023-01041-6.

## Introduction

Lung cancer is one of the most common types of malignant tumor [1, 2]. (Chemo)-Radiotherapy (CRT) is the standard treatment for locally advanced lung cancer. In radiotherapy, side effects still present great challenges in treatment management, while high-energy X-rays are killing the tumor tissues [3–6]. For example, acute radiation esophagitis (ARE), which occurs within 6 months after the first radiotherapy (RT) completion, is one of the major debilitating toxicities in patients with lung cancer following CRT [7]. The incidence rate of Common Terminology Criteria for Adverse Events (CTCAE) V4.0 grade  ≥ 2 ARE ranges from 30 to 50% [8], and is greater for higher radiation doses and the use of concurrent CRT (CCRT). ARE causes throat pain, dysphagia, and severe cases even cause complete obstruction, ulceration, or fistula formation in the esophagus [8]. Consequently, those symptoms, if not managed well, could seriously reduce the patient’s quality of life, incurring a large financial burden and a deteriorating prognosis [9]. More significantly, multiple studies have shown that the severe ARE contributes negatively to overall survival [10, 11]. Therefore, the pretreatment identification of the ARE using predictors will help physicians to better manage at-risk patients.

The reported key predictors to identify ARE in patients with lung cancer are inconsistent among studies. Palma et al*.* [12] found that the volume with the minimum dose of 60 Gy (V_60_) of the esophagus was a key dosimetric factor to predict ARE. Several dose−volume histogram (DVH) dosimetric parameters of the maximum dose, average dose, the dose with a volume of 5 cc (D_5 cc_) and the volume received dose larger than 20 Gy, 30 Gy, 35 Gy, and 40 Gy (that are V_20, 30, 35, 40_) of the esophagus [13] were recognized as predictors of ARE. Other studies also provided discrepant predictors from the esophagus DVH dosimetric parameters, such as V_50_, the equivalent doses, and D_2 cc_ [14–18]. One explanation for these discrepancies involves the different DVH factors believed to be associated with ARE, for example, D_5 cc, 10 cc_ used by Nieder et al*.* [13], and equivalent doses (EUD) adopted by Butof et al*.* [18]. Another explanation is the different three-dimensional (3D) dose distributions delivered by different RT techniques, for example, 3D conformal radiation therapy (3D-CRT) in the studies of Palma and Butof [12, 13, 18, 19], and intensity-modulated radiation therapy (IMRT) [14, 16, 20, 21]. Moreover, patients involved in each of the above-studied cohorts were treated with either an obsolete RT technique (i.e., 3D-CRT) or heterogenous RT techniques (i.e., 3D-CRT or IMRT). Therefore, patient cohorts using a uniform RT technique, especially advanced RT techniques of IMRT and volumetric modulated arc therapy (VMAT), are recommended to investigate ARE.

In terms of dosimetric predictors, the DVH or DVH-based parameters only characterize the dose information of the whole volume of interest (VOI), rather than representing the dose spatial pattern of the VOI. The dose spatial pattern is determined by the RT technique. To determine the dose spatial pattern, new features to describe the 3D-dose distribution, known as dosiomics, were proposed to predict radiation pneumonitis, and showed potential in clinical application [22]. In contrast, radiomics has recently been studied to investigate the intrinsic organ treatment response by characterizing the image-derived (magnetic resonance imaging (MRI)/computed tomography (CT)/positron emission tomography (PET), etc.) heterogeneous information of the VOI [23–27]. Overall, these three types of features, including DVH dosimetric, radiomic, and dosiomics features, capture comprehensively the heterogeneity of the VOI in dosimetry and imaging. Accordingly, several studies using a combination of two or three features have demonstrated an improvement in predictive model performance [28–30].

Despite the potential of using combination features, multi-omics features, including radiomics, dosiomics, and DVH dosimetric features, have been applied in a few studies to investigate ARE in patients with lung cancer treated with radiotherapy. Bourbonne et al*.* surveyed ARE in patients with lung cancer treated with RT VMAT by adopting the combined features, integrating the clinical, DVH dosimetric parameters, and radiomics [26]. To date, however, ARE in patients with lung cancer has not been investigated sufficiently with respect to treatment using IMRT.

The present study aimed to construct an integrated pretreatment ARE prediction model for patients with locally advanced nonsmall-cell lung cancer (LA-NSCLC) treated only with IMRT by adopting multi-omics features, based on the pretreatment planning CT image, RT structures, and the RT 3D dose distribution. Meanwhile, the complementary predictabilities of the three types of features were investigated by comparing the performances of the machine learning models constructed from single-mode features and multi-mode features.

### Materials and methods

**Fig. 1:**
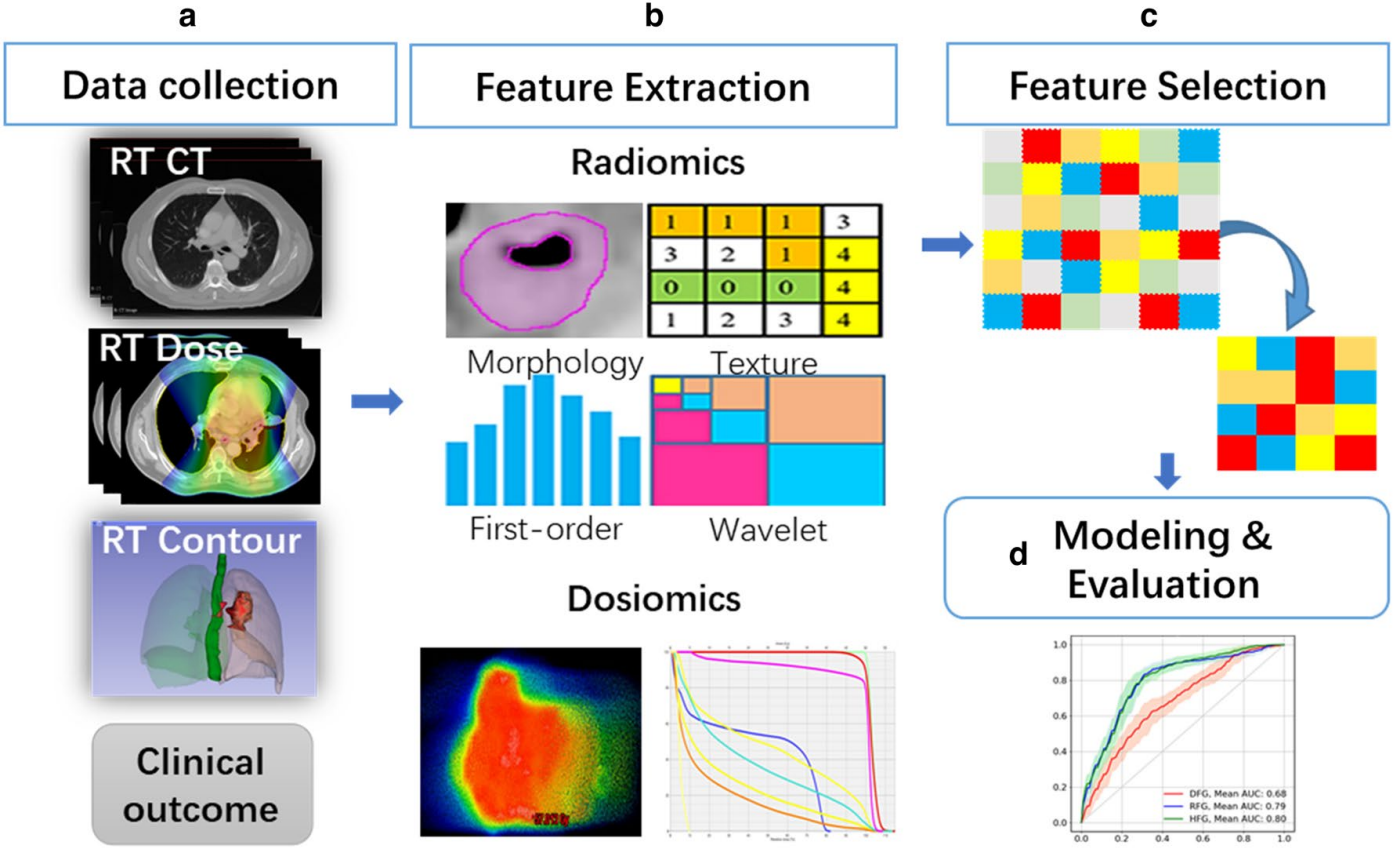
The framework of the classification model construction

In this study, model construction is consist of four parts (Fig. 1): (a) data collection, including images and clinical data; (b) feature extraction, multi-omics features (radiomics and dosiomics features) were extracted for the esophagus region based on the pretreatment planning CT image and the RT 3D dose distribution; (c) feature selection, a portion of the features were screened out using unsupervised and supervised methods, considering feature redundancy and relevance; and (d) modeling and evaluation, a classification model for ARE was built using the selected features and the regression classification algorithm; the model performance in the training and testing cohorts was evaluated using two metrics. The more details are shown below.

### Data collection

#### Patient data

The data for all patients with stage IIIA−IIIB LA-NSCLC treated in our institution from 2015 to 2019 were retrieved retrospectively in the study, using the following inclusion criteria: (a) age  > 18 years old at treatment, (2) diagnosed with lung cancer (nonsmall cell or small cell) with pathological findings, (3) treated with (chemo)-radiotherapy using 6 MV X-ray photon, (4) received IMRT in a curative manner, and (5) followed up for at least 6 months after treatment. The clinical factors, including patient’s age, gender, smoking status, TNM stage, pathology, RT prescription dose and fraction, treatment technology, and using chemotherapy or not, were collected. For chemotherapy, patients were given sequential or concurrent prescriptions. This study was approved by the ethical committee of the Affiliated Cancer Hospital of Zhengzhou University.

#### Radiation toxicities

The gradings of the acute radiotherapy toxicity, ARE, for all the patients were assigned by experienced physicians (≥ 5 years’ experience) following the CTCAE V4.0 protocol. The details of the grading criteria of CTCAE V4.0 protocol from grades 1 to 5 are as follows: (a) grade 1: asymptomatic, clinical or diagnostic observations only; intervention not indicated; (b) grade 2: symptomatic; altered eating/swallowing; oral supplements indicated; (c) grade 3: severely altered eating/swallowing; tube feeding, TPN or hospitalization indicated; (d) grade 4: life-threatening consequences; urgent operative intervention indicated; (e) grade 5: death. Acute toxicity was defined as toxicity events occurring within 6 months from the first radiotherapy treatment. All patients with their grading were summarized in the Table 1. In this study, we attempted to predict the severe ARE events with a grade ≥ 2.

**Table 1:** The patient’s number with each grade

Grade	1	2	3	4	5
Number	111	48	3	0	0

#### Image acquisition

All patients were immobilized with a vacuum cushion in a supine position, and underwent computer tomography (CT) scans using a 16-slice Brilliance Big Bore CT (Philips Medical System, Cleveland, OH, USA). The scanning parameters were as follows: voltage = 120 kV, X-ray tube current = 321 mA, thickness = 3 mm, spacing = 1.152 × 1.152 mm and with 512 × 512 pixels. In addition, the volume of interest (VOI) of the esophagus volume was segmented by physicians with at least 5 years of experience following the Radiation Therapy Oncology Group (RTOG) 1106 report [31]. It should be mentioned that the esophagus volume was contoured using mediastinal windowing on CT to correspond to the mucosa, submucosa, and all muscular layers out to the fatty adventitia. Besides, the esophagus contour begins at the level of the cricoid cartilage and continues on every CT slice including the gastroesophageal junction, until it ends at the stomach. To ensure the correction of segmentation, two physicians with at least 5 years of experience were involved in contouring esophagus volume, one physician for segmentation and another one for review and correction. The average volume of the esophagus in our data sets is about 37 cc.

### Feature extraction

Before feature extraction, CT images were resampled to a voxel size of 1 × 1 × 1 mm^3^. The types of radiomic features involved in the study were described in detail in the previous publication [32]. The only difference was in the bin counts, with the setting of [20, 30, 40, 50, 80, 100, 150, 200, 250, 300]. Besides, a threshold for the Hounsfield Unit (HU) for the range of [− 150, 180] was used to eliminate the nonesophagus region, such as air cavities. In total, 8990 radiomics features were extracted from the planning CT images within the esophagus volume.

In this study, dosiomics features consisted of three parts: (a) scale-invariant 3D dose moments [33, 34], 3rd order was chosen for three dimensions, resulting in 64 possible combinations. Except for the order of [0,0,0] with a constant value of 1, the other 63 combinations were contained in the study; b) DVH parameters [35, 36], i.e., Vx and Dx from the DVH curve, where Vx was the volume or % volume receiving a dose larger than x Gy, and Dx was the dose (Gy) to a relative volume of the esophagus; c) radiomics based on the 3D dose distributions [22], the original image type was used to extract radiomics features containing the first-order and high-order features. In total, 213 dosiomics features were acquired from the RT planning 3D dose distributions within the esophagus volume. In the feature calculation, we adopted our in-house developed Python-based platform based on the Python package *Pyradiomics* [37].

### Modeling and evaluation

#### Model construction

In this study, we constructed three classification models to predict whether a patient would develop severe ARE after IMRT up to the end of the follow-up period. The three classification models were generated using the clinical factors (CF model (CFM)), radiomics features (RF model (RFM)) only, dosiomics features (DF model (DFM)) only, and the hybrid features (HF model (HFM)), which combined both the clinical factors, radiomics and dosiomics features. All the model training and evaluations were performed using *Scikitlearn* in Python [38].

All the models were constructed following a standard procedure demonstrated in Fig. 1: (1) Features with high outcome relevance and low redundancy were selected under random patient subsampling; (2) All data were randomly divided into a training cohort (2/3) and a testing cohort (1/3) using 30 independent repetitions. Notably, the stratified sampling approach was used to keep the same event distribution between the training and testing cohorts. A series of classification models were trained in the training cohort using the selected features. (3) The final model was obtained with a comprehensive analysis of model performances in both training and testing under the condition of 30 repetitions.

**Fig. 2:**
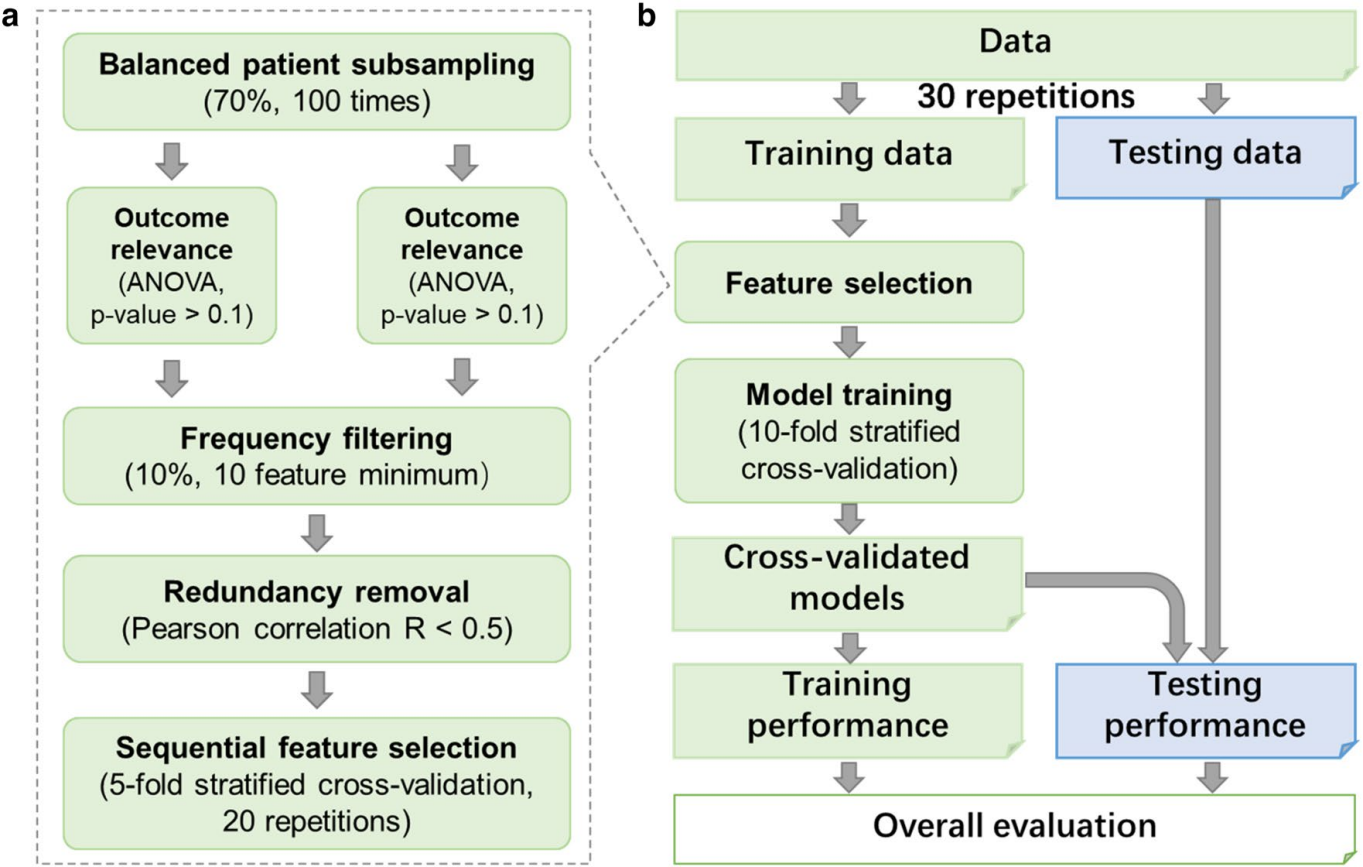
**a** The flowchart of feature selection. **b** The process of model construction

The process of feature selection was first proposed in a previous study [39]. The features were selected using a 100-time patient bootstrapping down-sampling method (see Fig. 2a ). At each sampling iteration, 70% of the entire patient cohort were randomly sampled and some features were filtered out using criteria of variance = 0, and *p* > 0.1 (*F* test). In the rest of the features, the 10% most frequent features (with a minimum feature number of 10) that were selected in the 100 down-sampling iterations were screened out. After that, the Pearson-*R* correlation test was used to remove correlated features using a threshold of 0.5 [40]. The final selected features were determined by the best performance of fivefold stratified validation with 20 repetitions among varied feature combinations.

All the models were trained using the selected features and the Ridge classifier, as shown in Fig. 2b. At each training iteration, the optimal model hyper-parameter was determined by grid-searching under tenfold cross-validation. In addition, easy ensembling with the 2/3 bootstrapping method was used to reduce the model bias from the imbalance of positive and negative cases. After that, the area under the curve (AUC) and accuracy (ACC) were used to evaluate the performance of each model in both the training and testing cohorts for each training–testing-split iteration. Finally, the average value and standard deviation of the AUC and ACC of the model series for 30 training–testing splits were calculated to characterize the overall predictability of the selected multi-model features.

In addition, a combined model was generated using the 30 models from the training–testing splits by means of an easy ensemble method. A nomogram using the optimal feature group were constructed for visualizing the classification model using a combined model.

## Results

### Data and features

Following the inclusion criteria, a total of 161 patients with LA-NSCLC diagnosed from 2015 to 2019 were included in the study. The patients’ characteristics are summarized in Table 2, and all of the characteristics showed a significant difference between the endpoints and the clinical factors.

**Table 2:** The overall characteristic information of all patients

Characteristics	Overall (161)	ARE (51)	*p* value
Gender			*p* < 0.001
Male (N/%)	142/88.2%	47/33.1%	
Female (N/%)	19/11.8%	4/21.1%	
Age, median (range)	62 (29–83)	–	*p* < 0.001
Pathology			*p* < 0.001
SCC (N/%)	104/64.6%	37/35.6%	
ADC (N/%)	51/31.7%	13/25.5%	
Others (N/%)	6/3.7%	1/16.7%	
RT Dose			*p* < 0.001
Median (range) $<$ 60 Gy	60 (45–70) Gy		
N/%	81/50.3%	24/29.6%	
Smoking			*p* < 0.001
Activity or former (N/%)	123/76.4%	38/30.9%	
Never (N/%)	38/23.6%	13/34.2%	
T Stage			*p* < 0.001
T1 (N/%)	10/6.2%	1/10.0%	
T2 (N/%)	70/43.5%	24/34.3%	
T3 (N/%)	35/21.7%	11/31.4%	
T4 (N/%)	46/28.6%	16/34.8%	
N Stage			*p* < 0.001
N0 (N/%)	9/5.6%	2/22.2%	
N1 (N/%)	4/2.5%	1/25.0%	
N2 (N/%)	85/52.8%	33/38.8%	
N3 (N/%)	63/39.1%	15/23.8%	
TNM			*p* < 0.001
IIIA (N/%)	54/29.2%	20/37.0%	
IIIB (N/%)	107/70.8%	31/29.0%	
Treatment technology			*p* < 0.001
SCRT (N/%)	65/40.4%	17/26.2%	
CCRT (N/%)	87/54.0%	31/35.9%	
RT (N/%)	9/5.6%	3/33.3%	
ARE (N/%)	51/31.7%	–	–

*SCC* Squamous carcinoma cancer, *ADC* Adenocarcinoma cancer, *SCRT* Sequential chemoradiotherapy, *CCRT* Concurrent chemoradiotherapy

As shown in the table, ARE toxicity rate in the whole dataset was approximately 31.7%. The average age was approximately 62 years old, with a standard deviation of 9.5 years, showing that almost all patients are from the senior group. Besides, we noticed that almost 66% of the patients had squamous carcinoma cancer. In addition, the majority of patients received the treatment comprising chemoradiotherapy.

After feature selection, 3, 6, 12, and 13 features were involved in the three models, using features of CF, DF, RF, and HF determined following the previously published procedure, as shown in Additional file 1: Table S1. As shown in the table, three clinical factors in the CFM are treatment technology, using chemotherapy or not. In addition, five dose features in the DF model belonged to spatial texture features, describing the spatial dose distribution. The other feature, V0.99, was from the DVH metric. The majority of the selected features in the RF model are related to the gray level matrix from the wavelet filter and log sigma [41], and only one selected feature was calculated based on the original CT images. In the HF model, all the selected features were from the radiomics features, i.e., none of dosiomics features and clinical factors were kept in the final optimal feature group. Thus, the eight selected features in the HF model were also adopted in the RF model.

### Model performance

**Fig. 3:**
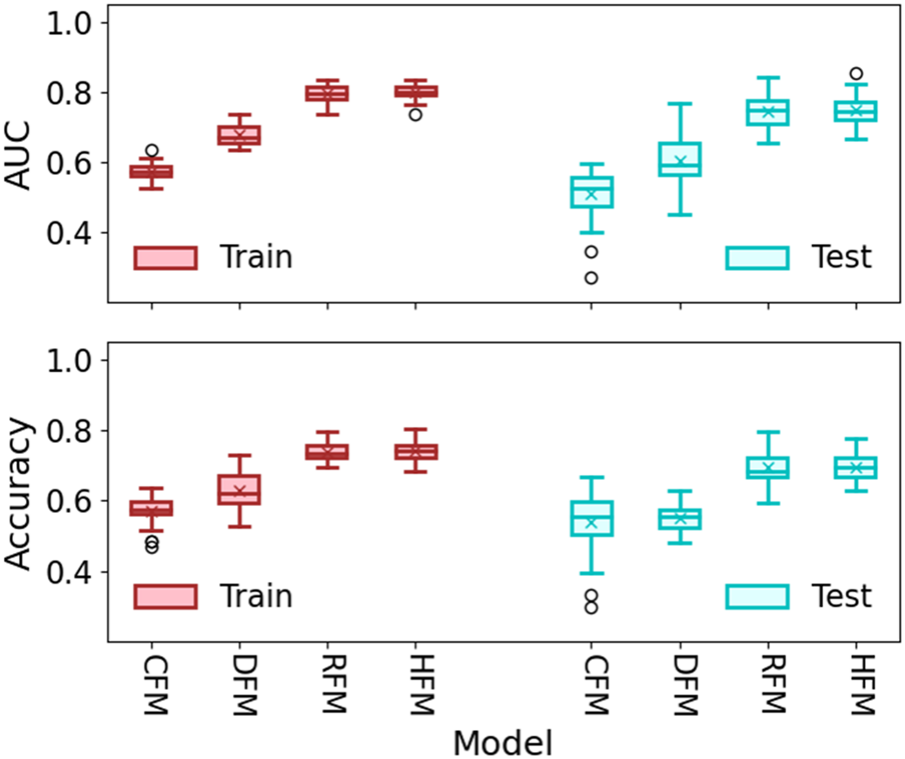
The comparison of four models using CF, RF, DF, and HF in the training and testing cohorts. The red and blue solid lines are the training and testing AUC, respectively. The shadow shows the standard deviation (STD), which the narrower of the shadow, the smaller of the STD. The upper and lower subfigures are the results of AUC and ACC, respectively

**Fig. 4:**
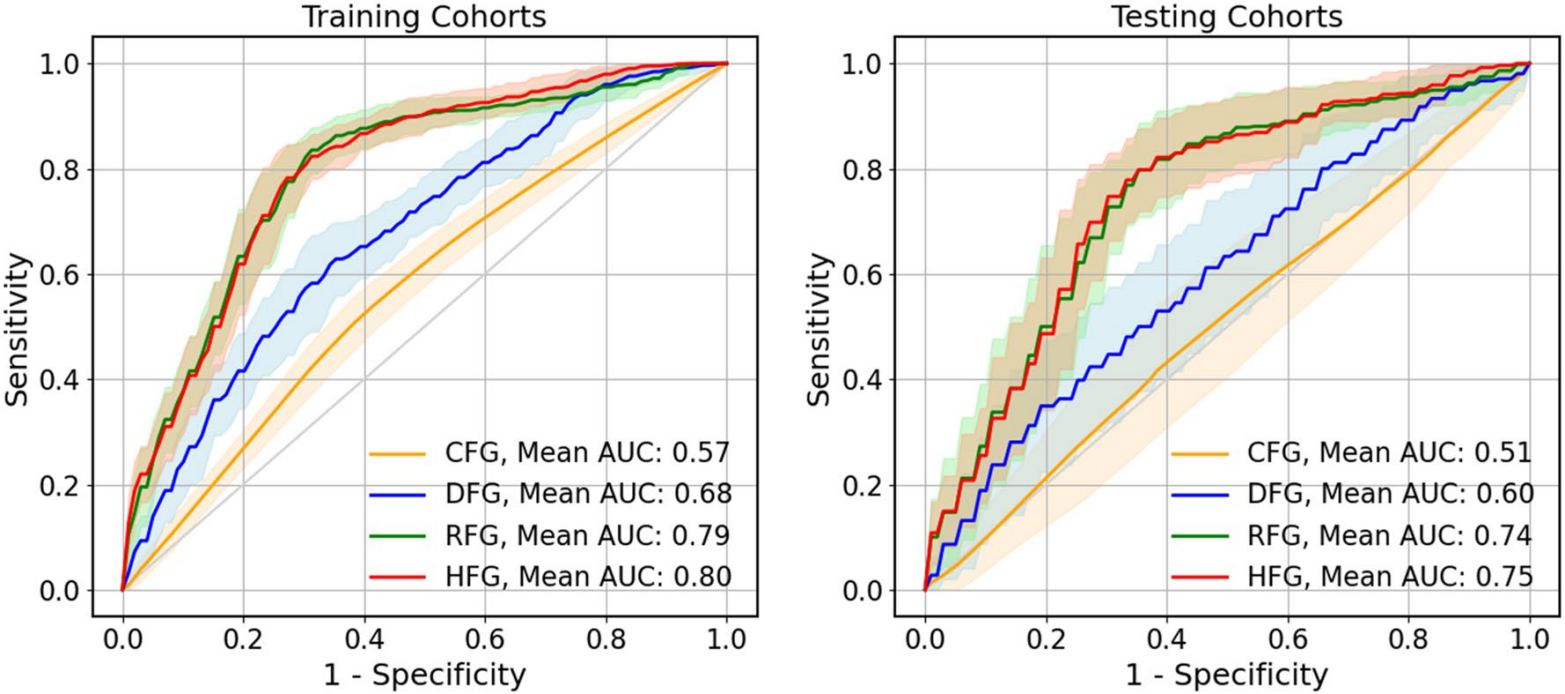
ROC curves for each model in the training and testing cohorts. The orange, blue, green, and red solid lines represent the results of CFG, DFG, RFG, and HFG

In the 30 training–testing splits mode, the average AUCs in the training and testing cohorts are shown in Fig. 3 and Table 3. From the figure, we observed that the models using the radiomics and hybrid features, i.e., RF and HF, achieved similar classification performance in training and testing, with AUCs of 0.796 ± 0.023 (95% confidence interval (CI [0.79, 0.80])/0.744 ± 0.044 (95% CI [0.73, 0.76]) and 0.801 ± 0.022 (95% CI [0.79, 0.81]) (*p* = 0.74), respectively. The model performance using CF and DF features showed a poorer predictive performance, with the training and testing AUCs of 0.573 ± 0.026 (95% CI [0.56, 0.58])/ 0.509 ± 0.072 (95% CI [0.48, 0.53]) and 0.679 ± 0.027 (95% CI [0.67, 0.69])/0.604 ± 0.041 (95% CI [0.53, 0.63]) compared with the above two models (*p* < 0.001), respectively. In addition, the receiver-operating characteristic curves (ROC) of three models were plotted in Fig. 4, and the nomogram using the combined models and the optimal feature group were shown in Additional file 1: Fig. S1. The probability of ARE can be read easily while the values of RadScore was calculated by the formula as shown in Additional file 1: Table S2. The points and total points in the Additional file 1: Fig. S1 are the normalization value in 0 to 100 using the RadScore. Two points can help user read the probability of ARE.

**Table 3:** The model performance in the multiple train−test splits model

Model	Metric	Training cohort ($\mu \pm \sigma$)	95% CI	Testing cohort ($\mu \pm \sigma$)	95% CI
CFM	AUC	$0.573\pm 0.026$	[0.56, 0.58]	$0.509\pm 0.072$	[0.48, 0.53]
	ACC	$0.571\pm 0.041$	[0.56, 0.58]	$0.538\pm 0.071$	[0.51, 0.57]
DFM	AUC	$0.679\pm 0.027$	[0.67, 0.69]	$0.604\pm 0.068$	[0.58, 0.63]
	ACC	$0.630\pm 0.049$	[0.61, 0.65]	$0.552\pm 0.037$	[0.54, 0.56]
RFM	AUC	$0.756\pm 0.023$	[0.79, 0.80]	$0.744\pm 0.044$	[0.73, 0.76]
	ACC	$0.740\pm 0.024$	[0.73, 0.75]	$0.695\pm 0.045$	[0.68, 0.71]
HFM	AUC	$0.801\pm 0.022$	[0.79, 0.81]	$0.747\pm 0.041$	[0.73, 0.76]
	ACC	$0.744\pm 0.030$	[0.73, 0.75]	$0.696\pm 0.041$	[0.68, 0.71]

*ACC accuracy, *$\mu$* average, *$\sigma$* standard deviation, CI confidence intervals*

The same results were found using the evaluation metric of accuracy, as shown in Fig. 3 and Table 3. The models using RF and HF have a similar performance with the training and testing ACC of 0.740 ± 0.02 (95% CI [0.73, 0.75])/0.695 ± 0.045 (95% CI [0.68, 0.71]) and 0.744 ± 0.030 (95% CI [0.73, 0.75])/ 0.696 ± 0.041 (95% CI [0.68, 0.71]), respectively. The CF and DF models had a poorer classification result, with the training and testing ACCs of 0.571 ± 0.041 (95% CI [0.56, 0.58])/ 0.538 ± 0.071 (95% CI [0.51, 0.57]) and 0.630 ± 0.049 (95% CI [0.61, 0.65])/ 0.552 ± 0.037 (95% CI [0.54, 0.56]), respectively.

## Discussion

In the present study, we investigated the radiation toxicity of the esophagus for patients with LA-NSCLC treated with CRT using the IMRT technique. Several models using single-omics (i.e., radiomics and dosiomics) and multi-omics (the combination of radiomics and dosiomics) were established to predict ARE with a grade  ≥ 2. The model performance revealed that ARE could be predicted using the pretreatment image and dose factors.

Using the single-omics feature, the performance of the model using radiomics was much better than that using dosiomics, under the multiple training–testing splits, with training average AUCs of 0.801–0.679 and testing average AUCs of 0.747–0.604 (*p* < 0.001). The same situation occurred for the model stability, with the SD of the training AUC being 0.023–0.027 and the SD of the testing AUC being 0.44 to 0.068, respectively. However, the multi-omics features could not improve the prediction performance for ARE compared with the radiomics features, showing similar classification results using RF and HF (*p* = 0.74). On the one hand, these results revealed that the radiomics features have an overwhelming correlation with ARE compared with dosiomics. These results were consistent with those of a previous study [26], in which all selected features in the model using multi-omics features (combining radiomics, dosiomics, and clinical factors) were chosen from among the radiomics features to predict ARE in patients with lung cancer treated with VMAT. None of the dosiomics features were adopted in the HF model. On the other hand, it might reflect the fact that the dosiomics features have limited predictability for ARE comparing to the radiomics features.

In the DF model, the majority of selected dosiomics features belonged to the spatial texture feature, except for *Esophagus_V0.99*, which is a one-dimensional dosimetric factor from the DVH. This is inconsistent with the previous studies using DVH metrics of *D*_*max, mean, 5* cc, 20, 30, 35, 40,50, 60_ and *V*_20, 30, 40, 50, 60_ [12, 14, 17, 18] to predict ARE. In both the RF and HF models, all utilized features were radiomics features obtained by characterizing the spatial texture information of the esophagus. This might reflect the fact that the radiosensitivity of esophagus tissue dominates the occurrence of acute radiation esophagitis, but there was no contribution was from the dose information. In addition, clinical factors were also investigated in our study, which is agreed with the findings of a previous study [42] that clinical factors had poor predictability for ARE. Besides, this study [42] also investigated the correlation between DVH dosimetric parameters (*D*_*mean,max,*_* V*_*40,50,60*_ of esophagus) and ARE, and demonstrated very limited classification performance, with an AUC range from 0.46 to 0.56 [42].

Our dataset showed radiation toxicity (i.e. ARE) of 31.7% when using IMRT radiotherapy only, which falls into the previously reported range of 30–55% [12, 13, 18, 26, 43] for ARE grades  ≥ 2. Apart from this, the incidence rates of ARE with a grade  ≥ 3 were about 3.7% in our dataset, which was lower than that of the previous studies (11.4% and 10.3%) [14, 42]. This might be caused using either a higher prescription dose [42] with a median dose of 66.6 Gy, or the traditional radiotherapy technique [14] of 3D-CRT. Both techniques result in a higher received dose for organs at risk, including the esophagus, in comparison with our study with a lower prescription dose (median dose of 60 Gy in our data set) or using the RT technique of IMRT. This demonstrated that the lower the dose received by the esophagus, the lower the incidence rates for severe acute radiation esophagitis  ≥ grade 3. Hence, even though the dose features were not adopted in the HF model or achieved poor prediction in the DF model, decreasing the dose in the esophagus region still can benefit the management of radiation esophagitis. Therefore, further investigation of other effective dose features is warranted in the future.

To verify the study’s conclusion [8], we also analyzed the incidence rate of ARE in different prescription dose regions and patients with or without the treatment of CCRT. We divided patients into three groups based on the prescription dose level: (a) low-dose group (prescription dose  < 60 Gy) with 52 patients; (b) median-dose group (prescription dose = 60 Gy) with 81 patients; and (c) high-dose group (prescription dose  > 61 Gy) with 28 patients. ARE incidence rates being 30.8%, 29.6%, and 39.3% in the low-dose, median-dose, and high-dose groups, respectively. The ARE rate in the high-dose group is higher than in the other two groups. However, there was no statistical difference among these three groups, with p values of 0.37 and 0.46 for high-median and high-low groups, respectively. Besides, we also separate all patients into two groups following the treatment with or without CCRT, a) the CCRT group (treatment using CCRT); (b) the other group (using the other treatments). ARE incidence rates are 35.6% and 24.2% for the CCRT and other groups, respectively. Even though there was a higher rate in the CCRT group, there was no statistical difference between the two groups (*p* = 0.24). Therefore, our data set agreed with the study’s conclusion [8] that the incidence rate of ARE is greater in higher radiation doses and the use of CCRT. Considering the statistical analysis results and the feature selection procedure, two factors of dose and treatment technology were not chosen in the final selected feature set.

In our study, the pretreatment radiotherapy image data were adopted for prior prediction of ARE. ARE prediction before RT treatment can aid clinical management by allowing more clinical care for patients at high risk. Clinical care could attenuate the side effects, i.e., ARE, and thus can improve the quality of life of patients in the mid-treatment and post-treatment phases to some extent. Consequently, it can benefit the overall survival of patients with locally advanced lung cancer [11].

The present study still has several limitations. First, we only adopted single-center retrospective small sample size radiotherapy data to construct the prediction model. It would be worth carrying out a comprehensive investigation of ARE using large cohort multi-center prospective medical information to verify our findings. Second, the study only employed the CT images and the RT 3D dose distributions. It is worth noting that multiple modality images, obtained by considering the MR, PET, and cone−beam CT (CBCT) images, have the potential for ARE prediction. Alam et al*.* [43] reported the potential predictability using CBCT and MR images by evaluating the esophagus volume changes in ARE. Another study demonstrated that the SUV_peak_ of PET images correlated significantly with the ARE [44]. Finally, the robustness of the radiomics features was not considered to improve the model generalizability. Several studies [45, 46] have investigated feature robustness using the image perturbation method, and the results showed that only some of the radiomics features in NSCLC cohorts are robust.

## Conclusions

Acute radiation esophagitis  ≥ grade 2 can be predicted using pretreatment RT image features for patients with lung cancer treated with IMRT. To predict ARE, the multi-omics features have similar predictability to radiomics features; however, dosiomics features have a limited classification performance.

## Supplementary Information

**Additional file 1: Table S1.** The selected features for the three feature groups. **Table S2.** The coefficient and interpolation of each feature for the nomogram. **Figure S1.** The nomogram of the easy ensemble model shows the ARE probability estimation.

## Data Availability

The datasets generated for this study are available on request to the corresponding author.
