# Supplementary material for: Multi-omics to predict acute radiation esophagitis in patients with lung cancer treated with intensity-modulated radiation therapy
Source: Eur J Med Res. 2023 Mar 19;28:126. doi: 10.1186/s40001-023-01041-6 (PMC10024847; doi:10.1186/s40001-023-01041-6)
Supplement: Supplementary file 1 — Additional file 1: Table S1. The selected features for the three feature groups. Table S2. The coefficient and interpolation of each feature for the nomogram. Figure S1. The nomogram of the easy ensemble model shows the ARE probability estimation. [file 40001_2023_1041_MOESM1_ESM.docx]

Supplementary Material

# Supplementary Table and Figure

Supplementary Table 1. The selected features for the three feature groups.

| Name | Features |
| --- | --- |
| CF | Treatment technology  FPS  chemotherapy |
| DF | Esophagus_dose_original_gldm_DependenceNonUniformity  Esophagus_dose_original_glszm_GrayLevelVariance  Esophagus_dose_moment_1_0_1  Esophagus_dose_original_glszm_SizeZoneNonUniformityNormalized  Esophagus_dose_original_glszm_SmallAreaLowGrayLevelEmphasis  Esophagus_V0.99 |
| RF | Esophagus_log-sigma-6-0-mm-3D_glszm_SmallAreaHighGrayLevelEmphasis_20_binCount  Esophagus_log-sigma-3-0-mm-3D_glszm_LowGrayLevelZoneEmphasis_20_binCount  Esophagus_wavelet-HLL_gldm_DependenceVariance_30_binCount  Esophagus_wavelet-LHH_firstorder_Minimum  Esophagus_wavelet-LHL_glszm_ZoneEntropy_80_binCount  Esophagus_wavelet-HLH_glcm_Imc2_30_binCount  Esophagus_original_glszm_LargeAreaHighGrayLevelEmphasis_150_binCount  Esophagus_log-sigma-3-0-mm-3D_firstorder_Minimum  Esophagus_wavelet-LLH_gldm_LargeDependenceHighGrayLevelEmphasis_50_binCount  Esophagus_wavelet-HLL_glszm_ZoneVariance_150_binCount  Esophagus_log-sigma-1-0-mm-3D_gldm_DependenceEntropy_50_binCount  Esophagus_wavelet-LHH_gldm_DependenceEntropy_30_binCount |
| HF | Esophagus_log-sigma-6-0-mm-3D_glszm_SmallAreaHighGrayLevelEmphasis_20_binCount  Esophagus_log-sigma-3-0-mm-3D_glszm_LowGrayLevelZoneEmphasis_20_binCount  Esophagus_wavelet-HLL_gldm_DependenceVariance_30_binCount  Esophagus_wavelet-LHH_firstorder_Minimum  Esophagus_wavelet-LHL_glszm_ZoneEntropy_80_binCount  Esophagus_original_glszm_LargeAreaHighGrayLevelEmphasis_150_binCount  Esophagus_log-sigma-3-0-mm-3D_firstorder_Minimum  Esophagus_wavelet-LLH_gldm_LargeDependenceHighGrayLevelEmphasis_50_binCount  Esophagus_wavelet-HLL_glszm_ZoneVariance_150_binCount  Esophagus_log-sigma-1-0-mm-3D_gldm_DependenceEntropy_50_binCount  Esophagus_wavelet-LHH_gldm_DependenceEntropy_30_binCount  Esophagus_wavelet-LHL_glcm_Id_20_binCount  Esophagus_wavelet-HHH_glcm_Imc1_80_binCount |

*Abbreviations:* glszm: Gray Level Size Zone Matrix, gldm: Gray Level Dependence Matrix, glcm: Gray Level Co-occurrence Matrix, V0.99: the volume larger than the doe of 99% maximum dose of esophagus.

*
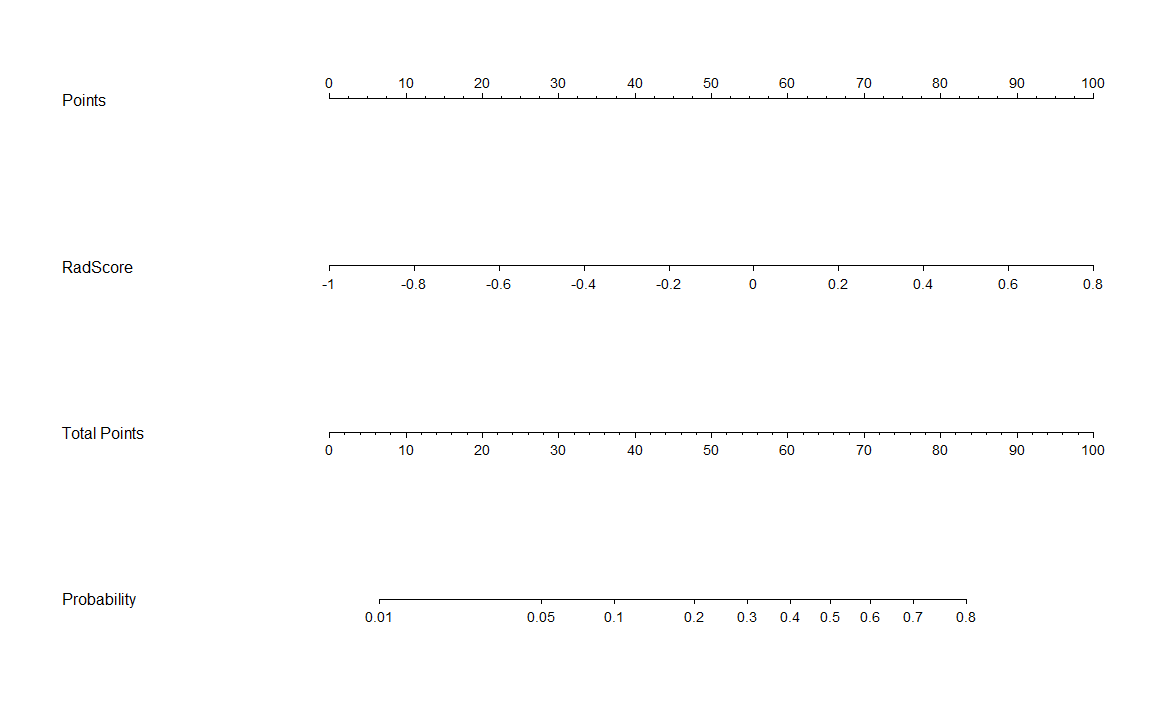
*

Supplementary Figure 1. The nomogram of the easy ensemble model shows the ARE probability estimation.

Supplementary Table 2 The coefficient and interpolation of each feature for the nomogram.

| Feature Name | Coefficients |
| --- | --- |
| Esophagus_log-sigma-6-0-mm-3D_glszm_SmallAreaHighGrayLevelEmphasis_20_binCount | -0.08698 |
| Esophagus_log-sigma-3-0-mm-3D_glszm_LowGrayLevelZoneEmphasis_20_binCount | -0.07911 |
| Esophagus_wavelet-HLL_gldm_DependenceVariance_30_binCount | -0.08197 |
| Esophagus_wavelet-LHH_firstorder_Minimum | -0.08468 |
| Esophagus_wavelet-LHL_glszm_ZoneEntropy_80_binCount | 0.077227 |
| Esophagus_original_glszm_LargeAreaHighGrayLevelEmphasis_150_binCount | -0.04154 |
| Esophagus_log-sigma-3-0-mm-3D_firstorder_Minimum | 0.031474 |
| Esophagus_wavelet-LLH_gldm_LargeDependenceHighGrayLevelEmphasis_50_binCount | -0.06561 |
| Esophagus_wavelet-HLL_glszm_ZoneVariance_150_binCount | -0.01109 |
| Esophagus_log-sigma-1-0-mm-3D_gldm_DependenceEntropy_50_binCount | 0.024429 |
| Esophagus_wavelet-LHH_gldm_DependenceEntropy_30_binCount | -0.03385 |
| Esophagus_wavelet-LHL_glcm_Id_20_binCount | 0.064799 |
| Esophagus_wavelet-HHH_glcm_Imc1_80_binCount | -0.04526 |
| Interpolation | -0.06186 |
